# Inequity in National Institutes of Health Predoctoral Fellowships, 2001-2020

**DOI:** 10.1001/jamanetworkopen.2022.38600

**Published:** 2022-10-26

**Authors:** Mytien Nguyen, Nghia D. Nguyen, Sarwat I. Chaudhry, Mayur M. Desai, Jose E. Cavazos, Dowin Boatright

**Affiliations:** 1 MD-PhD Program, Yale School of Medicine, New Haven, Connecticut; 2 Program in Neuroscience, Harvard University, Boston, Massachusetts; 3 Section of General Internal Medicine, Department of Medicine, Yale School of Medicine, New Haven, Connecticut; 4 Department of Chronic Disease Epidemiology, Yale School of Public Health; 5 South Texas MSTP, University of Texas Health San Antonio, San Antonio; 6 Department of Emergency Medicine, New York Grossman School of Medicine, New York

## Abstract

This cross-sectional study examines trends in number of awards and funding of general and diversity F31 predoctoral fellowships from 2001 to 2020.

## Introduction

Although a diverse biomedical workforce leads to more research innovation and productivity,^1^ Black, Hispanic, and American Indian or Alaska Native trainees made up only 7.7%, 7.8%, and 0.5% of all doctoral degree recipients in 2019, respectively.^2^ Recognizing the importance of predoctoral training in students’ eventual pursuit of an academic career, the National Institutes of Health (NIH) provides considerable support for predoctoral trainees^3,4^ in the form of T32 traineeship, research assistantships, and individual fellowship (eg, the Ruth L. Kirschstein Predoctoral Individual National Research Service Award [NRSA] F31). Unlike traineeships and research assistantships, fellowships are awarded to individual students, are assigned to study sections, and undergo the rigorous NIH peer review process. NIH fellowship recipients are more likely to receive future research funding and faculty appointments compared with nonrecipients.^5,6^ The NRSA F31 fellowship is categorized into 2 awards: a general F31 mechanism for all students and a diversity F31 fellowship for students who identify as Black, Hispanic, or American Indian or Alaska Native, have a disability, or are from a socioeconomically disadvantaged background.^3^ However, little is known about temporal trends in funding for F31 general and diversity fellowships. To fill this knowledge gap, we examined trends in number of awards and funding of general and diversity F31 predoctoral fellowships over the past 20 years.

## Methods

In this cross-sectional study, we examined award and funding rates for NIH predoctoral F31 NRSA fellowships. Grants data were retrieved from the NIH RePORTER Tool for fiscal years 2001 to 2020. Data on graduate student enrollment was retrieved from the NIH Databook. This study was deemed exempt and participant consent was waived by the Yale School of Medicine institutional review board. General and diversity F31 fellowships were determined based on their respective program announcement numbers. Total research dollars were inflation-adjusted to 2020 values. Descriptive statistics and simple linear regression were used to describe trends in total new awards and spending. *F* test was used to determine whether linear regression slope was significantly greater than 0, with 2-sided *P* < .05 indicating significance. This study followed the STROBE reporting guideline. All analyses were performed in GraphPad Prism v9.2 (Dotmatics).

## Results

**Figure 1.**
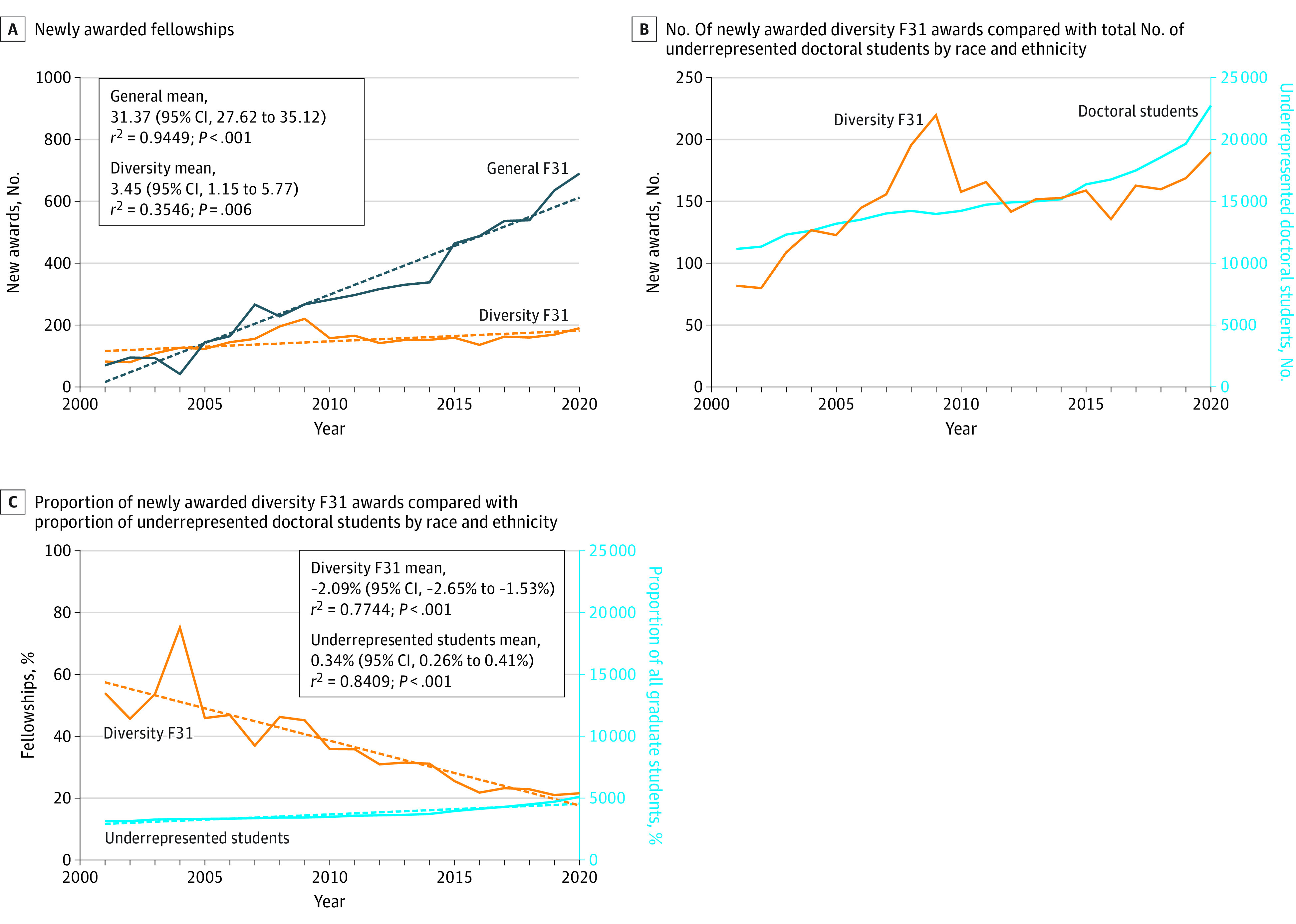
Trends in Annual Count of Newly Awarded F31 General and F31 Diversity Fellowships A, Count of newly awarded fellowships over time for general and diversity F31 awards with linear regression trends analysis. B, Count of newly awarded diversity F31 awards compared with total number of underrepresented by race and ethnicity (eg, Black, Hispanic, American Indian, Alaska Native) students. C, Percentage of newly awarded diversity F31 awards compared with proportion of underrepresented students. Dotted lines indicate the simple linear regression best fit line for each award type, and *P *values indicates statistical significance of slopes from 0.

Between 2001 and 2020, while the mean (SD) growth rate for general F31 fellowships was 31.37 (95% CI, 27.62-35.12) new awards per year, the growth rate for diversity F31 fellowships was 89% lower at 3.45 (95% CI, 1.15-5.77) new awards per year (*P* < .001) (Figure 1A). Notably, the number of new diversity F31 awards has remained stagnant since 2010 compared with a diversifying graduate student population (Figure 1B). Between 2001 and 2020, while the proportion of underrepresented graduate students increased by 0.34% (95% CI, 0.26%-0.41%) per year, the proportion of diversity F31 relative to general F31 decreased by 2.09% (95% CI, −2.65% to −1.53%) per year (*P* < .001) (Figure 1C).

**Figure 2.**
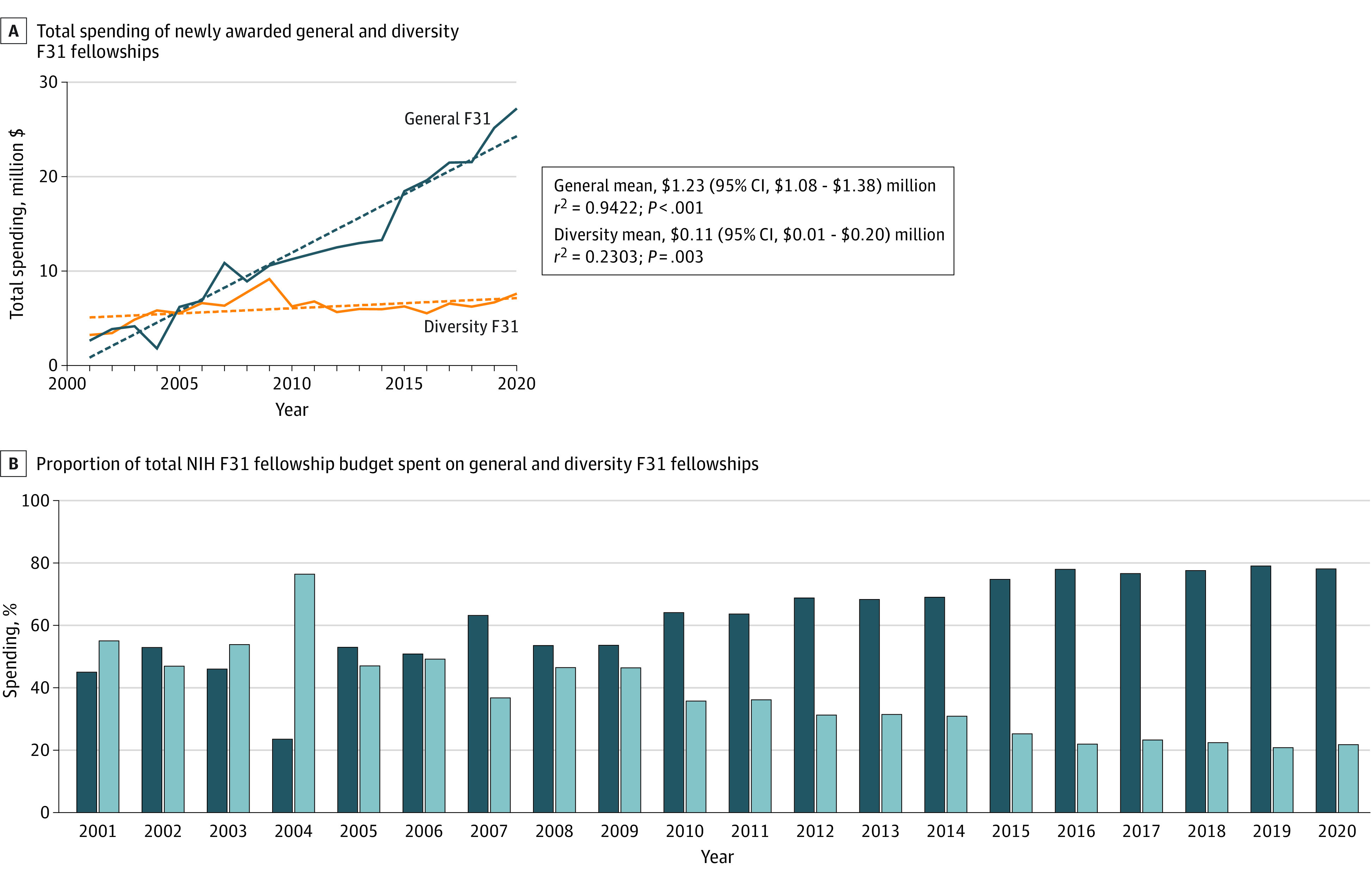
Annual NIH Spending on New General and Diversity F31 Fellowships A, Total spending (adjusted to 2020 dollars) of newly awarded general and diversity F31 fellowships over time with linear regression trends analysis; dotted lines indicate the simple linear regression best fit line for each award type, and *P* values indicate statistical significance of slopes from 0. B, Proportion of total NIH F31 fellowship budget spent on general and diversity F31 fellowships from 2001 to 2020.

Total NIH spending on F31 fellowships has increased more than 5-fold over time, from $5.9 million in 2001 to $34.8 million in 2020. During this time, funding to general F31 fellowship increased significantly ($1.23 million [95%CI, $1.08-$1.38 million]) compared with the diversity F31 fellowship ($0.11 million [95% CI, $0.01-$0.20 million]) (*P* < .001) (Figure 2A). The proportion of NIH funding toward diversity F31 relative to general F31 has decreased from 55.0% to 21.8% (Figure 2B).

## Discussion

In the last 2 decades, there has been a disproportionate growth of general NIH predoctoral F31 fellowships compared with diversity F31 fellowships despite an initial equal investment in general and diversity F31 fellowships, with a 90% greater increase for general F31 fellowship compared with diversity F31 fellowships. This widening gap may be reflective of a slower growth of applicants to diversity F31 fellowships, a decrease in award rate, or that underrepresented applicants are applying to the general rather than diversity F31 or are supported by other award mechanisms within or outside of the NIH. This study is limited by lack of data on numbers of applicants to F31 fellowships and personal information of applicants, which warrants future study.

Underrepresented biomedical science trainees face many challenges and barriers in their academic pursuits, including discrimination, racism, and lack of mentorship.^3^ Prestigious NIH fellowships, like the NRSA F31 fellowship, facilitate a path toward early career success for underrepresented trainees, increasing trainee’s likelihood of PhD completion, postdoctoral funding, and application to major research grants.^6^ Our data suggests that early gaps in predoctoral fellowships to underrepresented biomedical scientists may contribute to future inequity in research funding and career advancement.
